# Sialyltransferase Inhibitors for the Treatment of Cancer Metastasis: Current Challenges and Future Perspectives

**DOI:** 10.3390/molecules26185673

**Published:** 2021-09-18

**Authors:** Ser John Lynon P. Perez, Chih-Wei Fu, Wen-Shan Li

**Affiliations:** 1 Institute of Chemistry, Academia Sinica, Taipei 115, Taiwan; spperez@gate.sinica.edu.tw (S.J.L.P.P.); sandisefu@gmail.com (C.-W.F.); 2 Sustainable Chemical Science and Technology, Taiwan International Graduate Program, Academia Sinica, Taipei 115, Taiwan; 3 Sustainable Chemical Science and Technology, Taiwan International Graduate Program, National Yang Ming Chiao Tung University, Hsinchu 30010, Taiwan; 4 Department of Applied Chemistry, National Yang Ming Chiao Tung University, Hsinchu 30010, Taiwan; 5 Department of Chemistry, National Central University, Taoyuan City 32001, Taiwan; 6 Doctoral Degree Program in Marine Biotechnology, National Sun Yat-Sen University, Kaohsiung 804, Taiwan; 7 Ph.D. Program in Biotechnology Research and Development, College of Pharmacy, Taipei Medical University, Taipei 110, Taiwan; 8 Department of Medicinal and Applied Chemistry, College of Life Science, Kaohsiung Medical University, Kaohsiung 807, Taiwan; 9 Department of Chemistry, College of Science, Tamkang University, New Taipei City 251, Taiwan; 10 Biomedical Translation Research Center (BioTReC), Academia Sinica, Taipei 115, Taiwan

**Keywords:** sialyltransferase, hypersialylation, cancer metastasis, sialyltransferase inhibitors, drug design and development

## Abstract

Potent, cell-permeable, and subtype-selective sialyltransferase inhibitors represent an attractive family of substances that can potentially be used for the clinical treatment of cancer metastasis. These substances operate by specifically inhibiting sialyltransferase-mediated hypersialylation of cell surface glycoproteins or glycolipids, which then blocks the sialic acid recognition pathway and leads to deterioration of cell motility and invasion. A vast amount of evidence for the in vitro and in vivo effects of sialyltransferase inhibition or knockdown on tumor progression and tumor cell metastasis or colonization has been accumulated over the past decades. In this regard, this review comprehensively discusses the results of studies that have led to the recent discovery and development of sialyltransferase inhibitors, their potential biomedical applications in the treatment of cancer metastasis, and their current limitations and future opportunities.

## 1. Introduction

Cancer and the related phenomenon of metastasis continue to adversely affect both longevity and the quality of life of humans [1]. The leading cause of cancer-related mortality of patients (>90%) is metastasis and not the primary tumor. Evidence gained from in vivo studies shows that the pathogenesis of metastasis involves miscellaneous and characteristic gene-expression profiles that differ from those observed in normal organs and tissues [2,3,4,5]. Metastatic processes, which operate via lymph node and blood transmission routes, distribute cancer from the primary tumor site through basement membrane barriers and the extracellular matrix (ECM), and finally into secondary organs. Cancer metastasis involves multiple biological events including adhesion, degradation, and migration of tumor cells. Inhibition of any of these steps could block the entire metastatic course and, as a result, could serve as a therapeutic strategy for the treatment of cancer metastasis [6].

**Figure 1:**
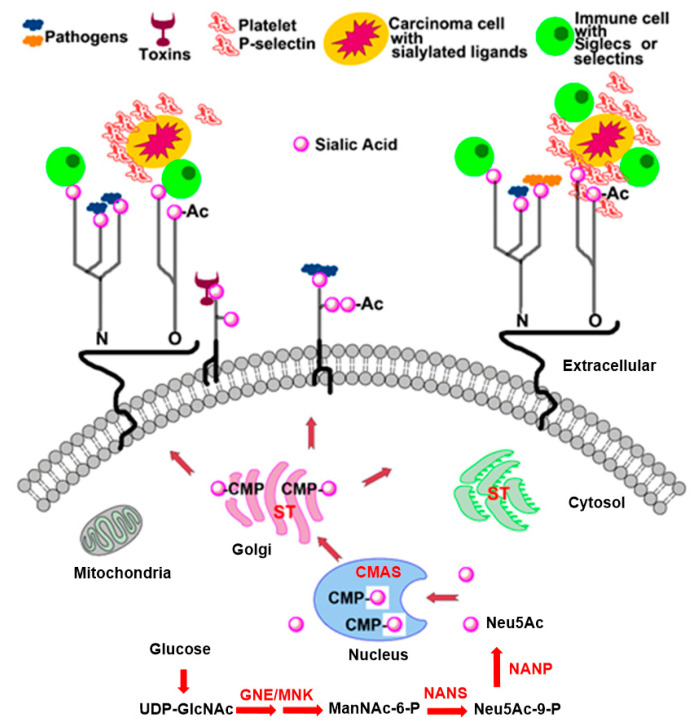
Illustration of the sialic acid metabolic pathway in cells and the pathobiological interactions of sialic acid residues on the cell surface.

**Figure 2:**
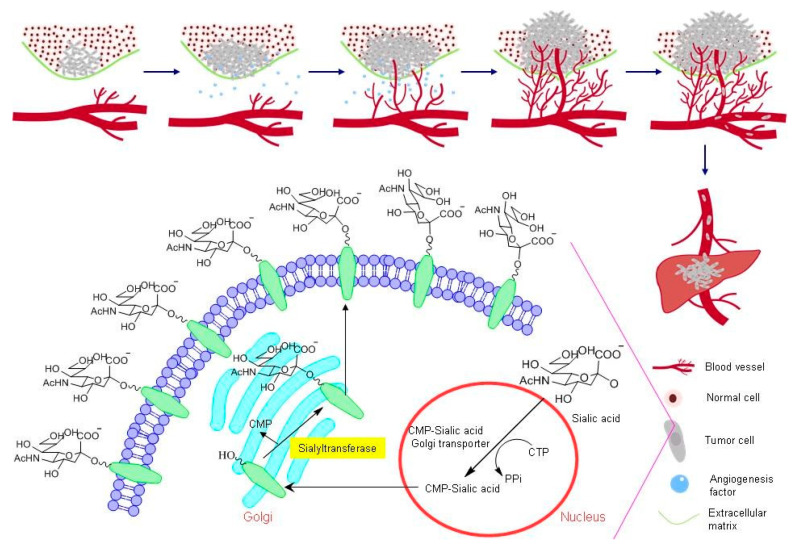
Overview of sialic acid metabolism in cancer cells and sialyltransferase-mediated hypersialylation of glycoproteins or glycolipids at the cell surface. The cartoon shows that alteration of sialylation on cell surfaces not only enhances tumor progression and invasion, but also facilitates angiogenesis and metastasis in the metastatic cascade.

Alteration of the extent of sialylation on the cell surface has been implicated in tumor cell progression, invasion, and metastasis. These processes are consequences of pathobiological interactions of negatively charged, cell surface sialic acid residues, connected to non-reducing termini of cell-surface glycans, with sites of pathogens, carcinoma cells or toxins (Figure 1) [7,8,9,10,11,12,13,14]. For instance, it has been clearly demonstrated that the existence of cell surface sialylated antigens is associated with cancer metastasis and invasion, and that the elevation in negative charge caused by sialic acid moieties on cell surfaces relates to the reduced adhesiveness of tumor cells [15,16]. In addition, an increase in the degree of sialylation often affects the stability, folding, and biological functions of proteins connected to important events including cell-cell interactions, cell migration, angiogenesis, and metastasis (Figure 2) [17,18,19].

Sialyltransferases (STs), a family of sialic acid dependent glycosyltransferases, catalyze the transfer of the sialic acid portion of cytidine monophosphate-*N*-acetylneuraminic acid (CMP-Neu5Ac) to hydroxyl groups on the ends of oligosaccharide chains of glycoproteins and glycolipids that contain either another sialic acid, galactose, or *N*-acetylgalactosamine via α2,3-, α2,6-, or α2,8-linkages [20,21]. Increased cell motility involved in the genesis of metastasis is a major factor for malignant neoplasm progress. Hypersialylation of terminal glycoconjugates in cell membranes plays an essential role in cell-cell adhesion, tumor cell metastasis and invasion. Hence, the discovery of potent ST inhibitors could represent an innovative and important strategy for the development of novel therapeutic agents for clinical treatment of cancer metastasis.

In this review, we comprehensively summarize and expound on the results of recent and relevant studies associated with the discovery, design, and development of ST inhibitors and the demonstration of the important roles they play in the regulation of cell surface sialic acids and its consequent impact on tumor cell invasion and metastasis.

## 2. Sialyltransferases and Cancer Metastasis

Twenty different human STs have been identified thus far and classified into four groups—ST3Gal, ST6Gal, ST6GalNAc and ST8Sia—according to the nature of their substrates (glycoproteins and glycolipids) and the type of linkage formed to the sialic acid (Neu5Ac) portion of CMP-Neu5Ac donor (α2,3-, α2,6-, and α2,8-), both of which play critical roles in molecular recognition and biological interaction by sialic acid binding proteins of pathogens (Figure 1) [21,22,23,24,25]. The family of ST3Gal (ST3Gal-I to VI) includes six beta-galactoside α2,3-sialyltransferases which catalyze formation of an α2,3-linkage between Neu5Ac and terminal galactose residues of either glycolipids or glycoproteins [26]. The ST6Gal (ST6Gal-I to II) group consists of two beta-galactoside α2,6-sialyltransferases which mediate the transfer of Neu5Ac residue to either the *N*-acetyllactosamine residue or type 2 disaccharides (Galβ1,4GlcNAc) of N- or O-linked oligosaccharides by formation of α2,6-linkages [27]. The family of ST6GalNAc (ST6GalNAc-I to VI) is comprised of six GalNAc α2,6-sialyltransferases that promote the introduction of Neu5Ac groups via α2,6-linkages to the *N*-acetylgalactosamine (GalNAc) residue of glycolipids (ST6GalNAc-III, V and VI) or O-glycosylproteins (ST6GalNAc-I, II and IV) [28]. Finally, the ST8Sia (ST8Sia-I to VI) group contains six α2,8-sialyltransferases, of which ST8Sia-II and ST8Sia-IV are polysialyltransferases. These enzymes mediate the transfer of the sialic acid portion of CMP-Neu5Ac to terminal sialic acid residues of glycoproteins and glycolipids via the generation of α2,8-linkages [29].

### 2.1. ST3Gal Family

Sialylation processes taking place in vivo are mediated by several glycoprotein and glycolipid specific glycosyltransferases, STs [14,24,30,31]. It is now evident that a member of the ST3Gal family, ST3Gal-I, acted as a tumor promoter in breast cancer and affected tumor development in a transgenic mice model [32]. Elevation of ST3Gal-I also led to the dominant expression of core 1 O-glycans on MUC1 [33,34]. A more recent article investigated the role of ST3Gal-I in ovarian cancer. Upregulation of ST3Gal-I was associated with enhanced cell growth, migration, and invasion. Moreover, overexpression of the enzyme conferred resistance to paclitaxel in vitro and increased tumorigenicity in vivo [35].

On the other hand, overexpression of ST3Gal-III modulated tumor progression, adhesion and migration in pancreatic cancer cell lines, and it augmented the metastatic potential in vivo [36]. Results from the same group demonstrated that pancreatic adenocarcinoma tissues expressed high levels of ST3Gal-III and ST3Gal-IV. The observed pathologies are consistent with data gained in studies with cell models that showed that expression of sLe^x^, E-selectin adhesion, migration, and metastasis formation are promoted in ST3Gal-III- and ST3Gal-IV-overexpressing cells [37,38]. Furthermore, ST3Gal-III modulated breast cancer cell adhesion and invasion by altering sLe^x^ expression, E-selectin binding capacity, and invasion-related protein expression including β1 integrin, MMP-2, and MMP-9 [39]. Similarly, altered expression of ST3Gal-IV led to the expression of sLe^x^ which then induced a more invasive phenotype in gastric cancer cells via activation of c-Met and its corresponding downstream molecular targets [40].

Increased expression of ST3Gal-VI was also identified in multiple myeloma cell lines and patients. Knockdown of the enzyme resulted in reduced cell adhesion and migration both in vitro and in vivo [41]. Interestingly, in hepatocellular carcinoma cells, ST3Gal-VI was negatively affected via microRNA (miRNA) regulation. It was showed that miR-26a deregulated ST3Gal-VI which in turn induced inhibition of cell proliferation, migration, and invasion in vitro. By targeting ST3Gal-VI, tumor growth was reduced in vivo through the suppression of the Akt/mTOR pathway [42]. In a similar manner, metastatic potential of hepatocellular carcinoma cells was suppressed by miRNA downregulation of ST6Gal-I expression [43].

High expression levels of ST3Gal-I and ST6Gal-I have been reported to be associated with non-melanoma skin cancer (NMSC). In comparison, it has been observed that skin tumors, such as squamous cell carcinoma, have increased levels of ST3Gal-I and ST6Gal-I associated with a greater potential for invasion and metastasis [44].

It has also been reported that overexpression of ST3Gal-III was responsible for lymph node metastases in FIGO IB1 cervical squamous cell carcinoma patients and that overexpression of ST6Gal-I played an important role when poor prognostic factors are present [45]. In a study investigating the functional effects of ST3Gal-III in cisplatin-resistant ovarian cancer cells, it was revealed that cisplatin-resistant HO8910PM cells with high invasive and metastatic capacity presented elevated ST3Gal-III expression. Upon ST3Gal-III knockdown, enhanced cisplatin-induced apoptosis was observed in ovarian cancer cells [46]. The same group demonstrated that a higher expression of ST3Gal-III was associated with enhanced paclitaxel resistance and reduced caspase-8/3 activity. Suppression of ST3Gal-III expression by siRNA interference increased caspase-8/3 protein levels and resulted in paclitaxel-induced apoptosis [47].

Moreover, elevated levels of ST3Gal-IV-generated α2,3 linked sialic acid residues are known to be present in gastric cancer tissues and overexpression of ST3Gal-IV may contribute to the formation of α2,3-linked sialic acid residues associated with invasion and cancer metastasis [48]. Measurement of ST expression in human hepatic cancer cell lines HepG-2 and SMMC-7721 revealed that ST3Gal-IV and ST6Gal-I were upregulated compared with control cell line and correlated with increased sialylation on cell membrane [49]. On the other hand, ST3Gal-II and ST3Gal-III mRNA levels were found to be elevated in oral cancer cells with advanced disease progression and metastasis [50].

It is also noteworthy that the mRNA levels of ST3Gal-I were found to be significantly higher in four malignant bladder cancer cell lines associated with the sialylation of T antigens that resulted in the increase of cancer progression and recurrence [51]. A good correlation has been found to exist between the elevated mRNA level of hST3Gal-V/hST6Gal-I and the high risk of pediatric acute leukemia. It has been suggested that for general systemic therapeutic purposes ST3Gal-V and ST6Gal-I RNA can be utilized as useful cancer markers [52].

### 2.2. ST6Gal Family

Earlier studies indicated that ST6Gal-I-catalyzed hypersialylation of β1 integrins promoted cancer progression by upregulating cell motility in vitro [13], and that the absence of hypersialylation favored carcinoma differentiation in vivo [53]. Since ST6Gal-I-dependent sialylation of the Fas death receptor provides protection against Fas-mediated apoptosis, downregulation of Fas α2,6 sialylation promoted apoptosis of colon carcinoma cells and even the expression of ras oncogene in cells [54]. Other investigations suggested that ST6Gal-I-mediated sialylation hampered binding of *N*-linked glycans to galectins, which are documented participants in various biological events including adhesion, angiogenesis, apoptosis, differentiation, inflammatory signaling, migration, survival, and tumor progression [55,56,57,58,59,60,61]. In addition, α2,6 sialylation of β1 integrins attenuated cell adhesion to galectin-3 and protected cells against galectin-3-mediated apoptosis [62]. In ovarian cancer cells, overexpression of ST6Gal-I promoted a metastatic phenotype, which was regulated by enzyme-mediated sialylation of β1 integrins in the extracellular matrix [63]. Higher ST6Gal-I mRNA levels from ovarian tumor samples correlated with lymphovascular invasion, poor prognosis, and distant metastasis [64]. In addition, ST6Gal-I played a major role in promotion of tumorigenesis and regulation of the stem cell phenotype in both normal and colon cancer cells where its overexpression was observed [65].

ST6Gal-I level was also significantly increased in non-small cell lung cancer (NSCLC) cells. Proliferation, migration and invasion capabilities of A549 and H1299 cells were suppressed in vitro by downregulation of ST6Gal-I and tumorigenicity of NSCLC cells in vivo was inhibited by ST6Gal-I silencing [66]. In human pancreatic ductal adenocarcinoma (PDAC) cells, ST6Gal-I was upregulated as a consequence of elevated *N*-acetylmannosamine levels associated with high fructose intake. In a fructose-responsive manner, elevated ST6Gal-I levels promoted distant cancer metastasis [67]. A very recent work revealed that ST6Gal-I-overexpressing pancreatic cancer cell lines exhibited higher EMT activation than a cell line with low endogenous ST6Gal-I. Forced ST6Gal-I overexpression of the other cell line activated EMT pathways, displayed enrichment in mesenchymal markers (N-cadherin, slug, snail, fibronectin) and suppressed epithelial markers (E-cadherin and occludin) [68].

Other findings further suggested that aberrant ST6Gal-I expression resulted in receptor sialylation and conferred cisplatin resistance in ovarian tumor cells. These observations highlighted why ST6Gal-I might be a potent therapeutic target in cisplatin-resistant tumors [69]. In a similar study, gefitinib-mediated cell death was suppressed in ovarian cancer cells by augmented activation of epidermal growth factor receptor (EGFR) which positively correlated with upregulation of ST6Gal-I expression [70]. Aside from EGFR, fibroblast growth factor receptors (FGFRs) have also been implicated in ovarian cancer malignancy. Likewise, it was established that FGFR1 sialylation increased phosphor-ERK1/2 and phosphor-focal adhesion kinase expression and in turn conferred chemoresistance and enhanced migration in ovarian cancer cells [71]. ST6Gal-I-mediated α2,6-sialylation was also reported to promote gastric cancer progression and confer chemoresistance [72]. Promotion of immune escape of hepatocarcinoma cells by ST6Gal-I-mediated sialylation was also demonstrated as a result of inhibiting T cell proliferation and upregulating CD145/MMP signaling pathways [73]. Another study pointed out the role of ST6Gal-I in hypoxia protection by augmenting HIF-1α accumulation in tumor cells [74].

Transforming Growth Factor-β (TGF-β), a cytokine that induces epithelial-mesenchymal transition (EMT) during cancer progression, was found to be promoted by overexpression of ST6Gal-I. By ST6Gal-I silencing, partial reversal of the basal mesenchymal phenotype of MDA-MB-231 breast cancer cells was observed [75]. Tumor necrosis factor (TNF) is another cytokine which mediates inflammation, cell differentiation, cell survival and growth, and apoptosis. By binding and activating tumor necrosis factor receptor 1 (TNFR1), cancer cell death or survival may be induced. It was reported that pancreatic and ovarian cancer cells with overexpression of ST6Gal-I exhibited resistance to TNF-induced apoptosis and reduced caspase-8/3 activation upon extended TNF treatment. This was attributed to the inhibition of TNFR1 internalization by the α2,6-sialylation by ST6Gal-I which resulted in cell surface localization [76].

Indeed, ever-expanding studies have established the significant role of ST6Gal-I in tumor-promoting processes including migration, invasion, immune escape, and chemoresistance. For more detailed summaries on the molecular mechanisms and events responsible for ST6Gal-I overexpression and its effects on cancer cell biology, recent reviews have been published [77,78]. Most of the studies in the ST6Gal family has been focused on only one subtype ST6Gal-I, thus the function and regulation of other ST subtypes remain unknown but may be important. In fact, a recent paper showed that ST6Gal-II downregulation inhibited cancer progression, cell adhesion and invasion, and suppressed ICAM-1, VCAM-1, CD24, MMP2, MMP9, and CXCR4 expression in breast cancer cells. All these findings implicated ST6Gal-II as another promising target for breast cancer treatment [79].

### 2.3. ST6GalNAc Family

For the ST6GalNAc family, past studies have shown that α2,6-ST (ST6GalNAc-V) mediated breast cancer metastasis to the brain [80]. The data indicated that ST6GalNAc-V promoted infiltration into the brain and that a brain sialyltransferase was responsible for regulating organ specific metastatic interactions through a cell surface glycosylation process. Interestingly, another work has shown that ST6GalNAc-V expression decreased adhesion or interactions between MDA-MB-231 breast cancer cells and the blood-brain barrier in an in vitro model [81].

Furthermore, the O-glycosylation pattern generated by ST6GalNAc-I observed in MDA-MB-231 breast cancer cells was accompanied by an increase of the sialyl-Tn antigen (sTn), Neu5Acalpha2-6GalNAc-O-Ser/Thr. The fact that expression of ST6GalNAc-I enhances tumorigenicity in SCID mice indicated that alteration of O-glycan biosynthesis affects the development of tumors [82]. In addition, the human ST6GalNAc-I and ST6GalNAc-II regulated the synthesis of the cancer-associated sTn antigen, which is associated with the aggressiveness and poor prognosis of carcinoma. Using a human gastric carcinoma cell line model, MKN45, stable transfection with either ST6GalNAc-I or ST6GalNAc-II induced carbohydrate antigens expression of sTn or Sialyl-6T, respectively. These results supported the proposal that ST6GalNAc-I plays an important role in sialylation of Tn antigen [83,84,85]. In another study, sTn-overexpressing cells were found to be protected against chemotherapeutics-induced cytotoxicity due to the reduction of galectin-3-binding sites in human gastric tumor samples. Knockdown of ST6GalNAc-I reversed the effect and restored drug sensitivity [86]. Similarly, siRNA silencing of ST6GalNAc-I inhibited gastric cancer cell proliferation, migration, and invasion in vitro, and prolonged survival of xenograft mice model [87]. Investigation of the effect of ST6GalNAc-II in follicular thyroid carcinoma has revealed that altered expression of the enzyme resulted in an invasive phenotype in vitro and in vivo by regulating the PI3K/Akt pathway [88].

### 2.4. ST8Sia Family

Human colorectal cancer progression was inhibited by miRNA downregulation of ST8Sia-I. In the study, ST8Sia-I expression negatively correlated with miR-33a/let-7e expression. Colorectal cancer cells with overexpressed ST8Sia-I have enhanced chemoresistance, proliferation, invasion, angiogenesis in vitro, and tumor growth in vivo. ST8Sia-I knockdown resulted in the reversal of the effects [89]. In a comprehensive transcriptomic analysis, it was identified that high ST8Sia-I expression was associated with poor survival among breast cancer patients [90]. Moreover, upregulation of ST8Sia-I conferred chemoresistance in triple negative breast cancer (TNBC) cells. Inhibition of ST8Sia-I downregulated the FAK/Akt/mTOR and Wnt/β-catenin signaling pathways and improved chemosensitivity [91,92]. Sarkar and others also reported that ST8Sia-I (GD3 synthase) facilitates EMT by activating c-Met and promoting migration, invasion, and in vivo metastasis of breast adenocarcinoma cells [93].

Altered expression of polysialic acid has been implicated in the progression of many tumors wherein it adorns the surface of neural cell adhesion molecule (NCAM) and facilitates cell migration and invasion. Polysialic acid-NCAM expression correlated with poor clinical prognosis and a more invasive phenotype in various cancers. The synthesis of polysialic acid is mediated by ST8Sia-IV and ST8Sia-II. Hence, selective inhibition of polySTs poses an opportunity to suppress tumor invasion and metastasis [94,95,96]. As a proof of concept, cytidine monophosphate (CMP) inhibition of ST8Sia-II reduced tumor cell surface polysialic acid expression and tumor cell migration [97].

During the past years, immense biological evidence has been amassed to support the implication of hypersialylation in the disease progression of an extensive range of carcinomas (summarized in Table 1). For the reader’s reference, several most recent review papers which provide a more comprehensive discussion on the involvement of sialylation on cancer progression and metastasis have been published [98,99,100]. All these evidence highlight the promising and continuing opportunity of sialyltransferase inhibitors as novel therapeutics for cancer metastasis.

## 3. Development of Small Molecule Sialyltransferase Inhibitors

Given the massive collection of biological evidence that they are associated with tumor progression, adhesion, and migration in human cancer cells, sialyltransferases and their sialylation processes serve as potentially significant targets in strategies designed to uncover and develop treatments for cancer metastasis. Several types of ST inhibitors, developed for this purpose, have structures that are closely related to the sialylation donor and acceptor substrates, or donor-acceptor bisubstrates, transition-state analogues, natural products, bile acids, flavonoids, among others. It is important to note that only few of the currently reported ST inhibitors are cell-permeable and that the lack of membrane permeability has hampered the biological and clinical applications of some of the most potent ST inhibitors. Recently, efforts of several groups have led to breakthroughs in the design of cell-permeable ST inhibitors, whose potential applications to cancer treatment (especially for cancer metastasis) are particularly attractive. Some of these investigations have been previously reviewed [101,102,103], but in this present review, we expound on the opportunities and challenges in the recent development of carbohydrate and non-carbohydrate ST inhibitors as well as their potential for the treatment of cancer metastasis.

### 3.1. Carbohydrate or Sugar-Based Inhibitors

**Figure 3:**
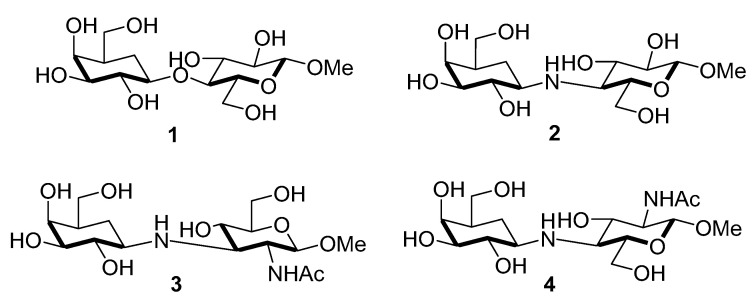
Chemical structures of 5a’-carbaoligosaccharides as acceptor mimics **1–4**.

Some inhibitors belonging in this huge family include substances that bind to the active sites of STs in competition with natural substrates but cannot undergo sialylation because they lack the required acceptor groups. These compounds have been developed for several mammalian and bacterial STs, which exhibit strict specificity for substrates containing terminal *N*-acetyllactosamine or lactose moieties at the non-reducing ends of glycoconjugates [104,105]. Ogawa and coworkers found that the methyl 5a’-carbadisaccharides **1**–**4** (Figure 3), in which the monosaccharide units are linked via ether or amine bridges, possessed considerable inhibitory activities (IC_50_ = 185–419 μM) towards recombinant α2,3-sialyltransferases using 4-methylumbellipheryl-labeled LacNAc as the acceptor substrate (Table 2) [106]. The inhibitory activities of **1**–**4** towards rat liver α2,6-sialyltransferases were also determined and shown in Table 2.

**Figure 4:**
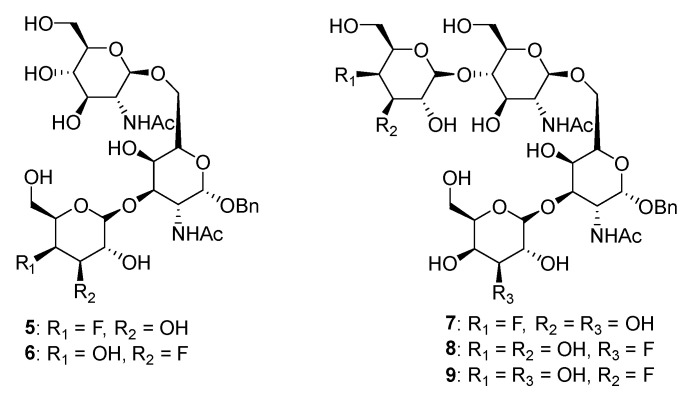
Structures of fluorinated mucin core 2 branched oligosaccharides **5–9**.

Matta’s group designed and synthesized several fluorinated mucin core 2 branched oligosaccharides (**5**–**9**, Figure 4) [107]. The results demonstrated that compound **7** had a modest inhibitory activity (inhibition constant, K_i_ = 1.9 mM) against cloned α2,6-(N)-ST (rST6Gal-I) but not α2,3-(N)-ST (rST3Gal-III). Interestingly, other substances in this group did not display inhibitory properties against STs. Moreover, **8** and **9** served as good acceptor substrates of α2,6-(N)-ST/α2,3-(N)-ST and cloned α2,3-(O)-ST/prostate cancer cell LNCaP α2,3-(O)-ST, respectively. Their findings indicated that the position of fluorine substitution on the mucin core 2 branched oligosaccharides affects the nature of the carbohydrates-enzyme interactions and, as a result, determines whether these substances can serve as ST substrates (acceptor) or inhibitors. To this date, no recent acceptor analogue ST inhibitors were reported.

**Figure 5:**
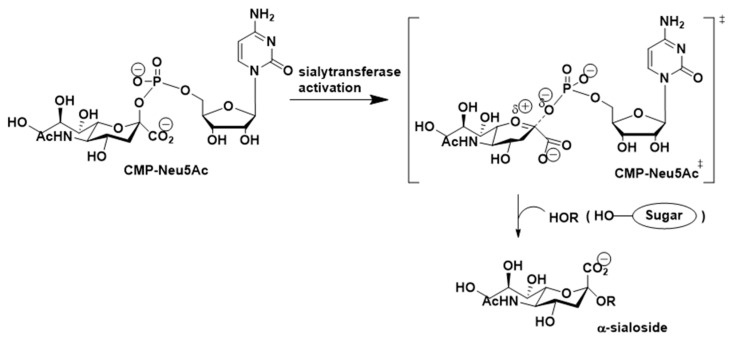
Proposed mechanism of sialyltransferase-catalyzed reactions.

Several studies conducted by Horenstein and Schmidt aimed at gaining insights into the detailed mechanism of the ST-promoted sialylation reactions. The observations made through this effort suggested that sialyltransferase-catalyzed reactions of the sialyl donor, (CMP-Neu5Ac), proceed through a transition state in which the leaving group (CMP) departs before bond formation with the incoming hydroxyl nucleophile. The transition state (Figure 5) for this process is believed to display oxocarbenium ion-like character with the carbohydrate ring existing in a distorted half-chair conformation and possessing a considerable amount of positive charge on the anomeric carbon atom [108,109,110,111,112,113].

Building upon this idea, several transition state analogues have been designed and tested as ST inhibitors. Most of these substances—which contain (i) a planar anomeric carbon, (ii) an increased distance between the CMP leaving group and the anomeric carbon, and (iii) at least two negative charges close to the site that mimics glycosylation cleavage—exhibited high affinities to sialyltransferases [113,114,115,116,117,118,119,120].

**Figure 6:**
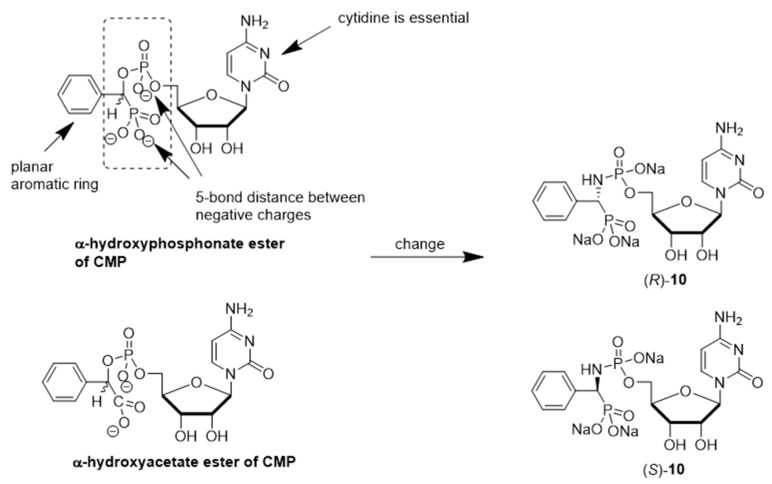
Structures of aromatic phosphoramidate CMP derivatives (*R*)-10 and (*S*)-10.

Schmidt and coworkers prepared the enantiomeric phosphoramidate-linked substances, (*R*)-10 and (*S*)-10 (Figure 6), which possess *N*-(α-phosphoryl)-phosphoramidate nucleosides and aromatic moieties, as potential ST inhibitors. These phosphoramidates mimic the CMP-Neu5Ac portion of the transition state structure in the sialylation reaction [121]. The results of this study showed that (*R*)-10 and (*S*)-10 are both competitive inhibitors of α2,6-ST with respective K_i_ values of 68 ± 24 and 140 ± 30 μM. Moreover, the presence of *N*-(α-phosphoryl)-phosphoramidate nucleoside and aromatic moieties in (*R*)-10 resulted in a binding affinity towards rST6Gal-I that is comparable to that of the neutral substrate CMP-Neu5Ac (K_m_ = 46 ± 7 μM).

Cytidine diphosphate (CDP), a potent competitive inhibitor (K_i_ = 10 μM) of sialyltransferases, mimics the CMP portion of the donor substrate CMP-Neu5Ac. Despite its potency, CDP and its analogues have not been subjected to thorough biological studies as inhibitors of STs [114]. On the other hand, the Fukuda group synthesized donor analogues 5-methyl CMP and 2′-O-methyl CMP as polysialyltransferase inhibitors. Their results revealed that 2′-O-methyl CMP strongly inhibited ST8Sia-IV, ST8-Sia-II, and ST8Sia-III. Upon treatment with the inhibitors, polysialic acid expression on the cell surface was reduced [97,122]. Our efforts identified a group of 5′-triazole nucleosides that are analogues of CMP using a substrate-based drug design approach. These substances, containing a unique 1,2,3-triazole subunit, were synthesized using Cu(I)-catalyzed Huisgen 1,3-cycloaddition reactions (click chemistry) [123,124]. Among the CMP analogues tested, most exhibited low inhibition of rST3Gal-I (Table 3). Only compound **11** was found to be more potent with an IC_50_ of 37.5 μM which suggests that the aromatic functionality and the cytosine group are required for better binding in the enzyme active site.

To gain a more thorough understanding of how binding of sugar-based ST inhibitors is governed by molecular shape, charge, H-bonding, and hydrophobic interactions, Zou and coworkers designed three types of substances including substrate mimics containing the 2-deoxy-2,3-dehydro-acetylneuraminic moiety and derivatives of aryl sulfonamides attached to cytidine [125]. One subgroup of these substances including **13**–**14** (Table 4) are substrate mimics in that they possess a non-hydrolysable and uncharged 1,2,3-triazole moiety as the linker rather than a phosphate group [126,127]. The second group of ST inhibitors includes **15**–**21**, all of which has a 2-deoxy-2,3-dehydro-acetylneuraminic moiety attached to cytidine through different carboxylic acid and amide linker groups [128]. The third type of substances represented by **22**–**39** possess an aryl sulfonamide group that replaced the sialylphosphate group in the CMP-Neu5Ac structure. All these designs are driven by the fact that the charged phosphate linker provides poor cellular permeability and instability towards phosphatases. Among these substances, **35** was observed to have the highest inhibitory activity against Campylobacter jejuni sialyltransferase (CJ ST) Cst 06 with a K_i_ value of 87 μM [129]. Other members of this series except for **13**, **14**, **34**, and **37** displayed low activities. It is however noteworthy that the first group of compounds are competitive inhibitors of CJ ST, while members of the other two subfamilies inhibit in a non-competitive fashion. These observations suggest that the charge and hydrophobic character, rather than molecular shape and H-bonding interactions, contribute more to the tight binding of these compounds against CJ ST. Therefore, it seems that a more practical way to improve the drug-like properties of these inhibitors is not to totally eliminate the charge but to mask it temporarily as a pro-drug.

**Figure 7:**
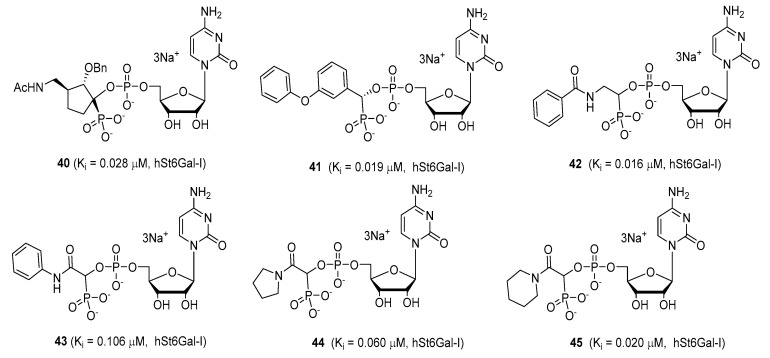
Structures of recently reported CMP-Neu5Ac transition state analogues [119,130,131].

Another set of cyclopentane-based transition state analogues were designed and prepared by Ye and others [130]. Their design is based on the idea that the cyclopentane skeleton in its puckered conformation may mimic the planar structure of the donor in the transition state. Hence, in this study, the Neu5Ac moiety in the donor was replaced by cyclopentyl α-hydroxyphosphonates and the substituent effects on the five-membered ring were analyzed. A potent inhibitor **40** with a K_i_ of 0.028 ± 0.006 μM was identified (Figure 7), slightly weaker than the previously reported 3-phenoxybenzyl-based inhibitor [119] from Schmidt’s group **41** which has a K_i_ of 0.019 ± 0.0065 μM [130]. A more recent work by the same group explored the attachment of an amide group to CMP to mimic the geometry and charge distribution in the transition state. This new set of compounds **42**–**45** possessed excellent α2,6-sialyltransferase inhibitory activities (Figure 7), which suggests that the amide group can be a facile and interesting structural isostere for transition state ST inhibitors [131].

Even though the most potent ST inhibitors reported are these transition state analogues, such compounds suffer from low lipophilicity and poor pharmacokinetic properties and are generally synthetically challenging. Skropeta and others took advantage of the availability of the structure of human ST8Sia-III to perform a structure-based computer-aided design [132]. Computer simulations revealed that among the transition state-based inhibitors studied, there was no significant difference in their calculated binding affinities upon substitution of cytidine with a uridine moiety as well as between R and S diastereomers. Molecular dynamics simulations of proposed carbamate- and triazole-linked analogues showed similar binding to the enzyme’s active site relative to other reported analogues. Such proposed neutral linkers are attractive for they may address the poor pharmacokinetic properties brought about by the phosphodiester linkage in previously developed ST inhibitors and may even offer potential selectivity towards hST8Sia-III [132]. The same group performed a similar study using the crystal structure of human ST6Gal-I [133]. In this work, it was demonstrated that compounds with a neutral carbamate or triazole linker maintained key interactions in the active site of the enzyme. Interestingly, the neutral linkers were slightly more favorable than the charged phosphodiester moiety, suggesting that a carbamate or 1,2,3-triazole group may serve as a bioisostere of the phosphodiester group in the development of novel inhibitors. Thus, the same group recently published the synthesis and ST6Gal-I inhibition studies of 24 carbamate-linked uridyl-based compounds [134]. Among them, five promising compounds (**46**–**50**) (Table 5) exhibited K_i_’s in the range of 1–20 μM. Given the potency of these ST inhibitors in vitro, it would be very interesting to understand their mechanism of action and investigate their functional effects in vivo.

**Figure 8:**
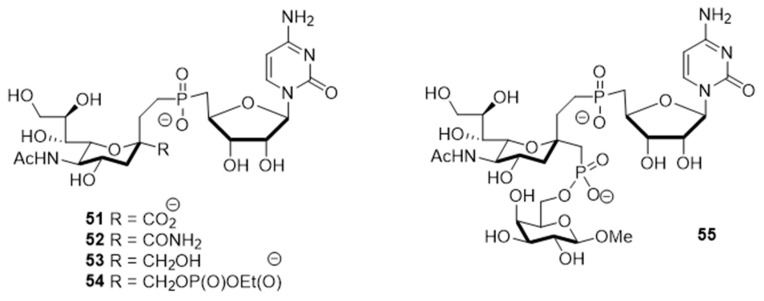
Structures of bisubstrate analogues **51**–**55**.

The utility of bisubstrate-type substances, such as those that contain both donor (CMP-Neu5Ac) and acceptor (*N*-acetyllactosamine, LacNAc) components, as sialyltransferase inhibitors was already recognized earlier [135]. Izumi and coworkers designed the bisubstrate analogue **55**, containing the donor-like substrate (CMP-Neu5Ac mimic) and the acceptor substrate (galactose) [136]. Four donor analogues, **51**–**54**, possessing the partial structure of the bisubstrate analogue, were used as controls in this structure-activity relationship study. The unique structural feature in each analogue is an ethylene group that replaced the exocyclic anomeric oxygen of CMP-Neu5Ac. Donor analogues **51**–**54**, possessing different replacements for the C-1 carboxylate group of the Neu5Ac moiety (carboxyamide, hydroxymethyl, or methylene phosphate), displayed decreased inhibitory activities against rat recombinant α2,3- and α2,6-ST. Among these substances, cytidin-5′-yl sialylethylphosphonate **51** gave moderate inhibitory activities with respective IC_50_ values of 0.047 and 0.34 mM against two rat recombinant α2,3- and α2,6-ST, which are 10−100-fold lower than those of **52** and **53** (Figure 8 and Table 6). Unlike **51**, the bisubstrate analogue **55** has less potent inhibitory activities against rat recombinant α2,3- and α2,6-ST (IC_50_ = 1.3 and 2.4 mM, respectively). As evident from inspection of the data displayed in Table 6, insertion of a charged group and extension of the C-1 carboxylate did not lead to the enhancement of inhibitory potency towards STs. Therefore, refinement of this strategy for designing bisubstrate inhibitors remains necessary.

As depicted in Figure 5, transfer of sialic acids to terminal positions of oligosaccharide chains of glycoconjugates catalyzed by ST is believed to proceed through a mechanism involving an oxocarbenium ion-like transition state. This mechanistic proposal suggests that incorporation of fluorine on the pyranose ring of sialic acid would give fluorinated CMP-sialic acids that may be weak ST substrates but perhaps good ST inhibitors [137,138]. This proposal is based on the likelihood that strongly electronegative substituents such as F, incorporated especially at C-3 position of the ring, would destabilize the forming cationic transition state and result in a decrease in substrate activity without affecting binding to STs. The results of studies validated this proposal by showing that commercially available CMP-3FNeu5Ac inhibited recombinant α2,6-ST and was utilized as a chemical probe for mechanistic, kinetic, and structural studies of STs and related enzymes such as sialidases [139,140].

**Figure 9:**
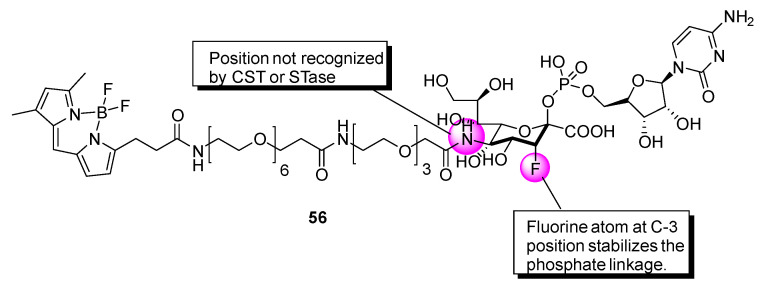
Chemical structure of **56**.

Further investigations have been carried out to expand the mechanistic understanding of the CMP-sialic acid transporter (CST)-promoted transport of CMP-sialic acid from the cytoplasm into the lumen of the Golgi apparatus [141]. Kanie and coworkers conducted studies with the fluorescent CMP-3′′-F-Sia derivative **56** (Figure 9), which is composed of a CMP-β-3′′-F-Sia moiety linked to a fluorescent tag [142]. This probe was found to have an inhibitory potency (K_i_ = 31.7 μM) against rST3Gal-III relative to that of the parent compound lacking the fluorophore (K_i_ = 5.7 μM, [rST6Gal-I]) [139,143,144]. Uptake of **56** into isolated Golgi vesicles of rat liver was determined using a competitive import test with CMP-Sia, which retarded the accumulation of **56**, suggesting that **56** can be employed as a substrate for CST in studies designed to gain information about glycan processing events.

**Figure 10:**
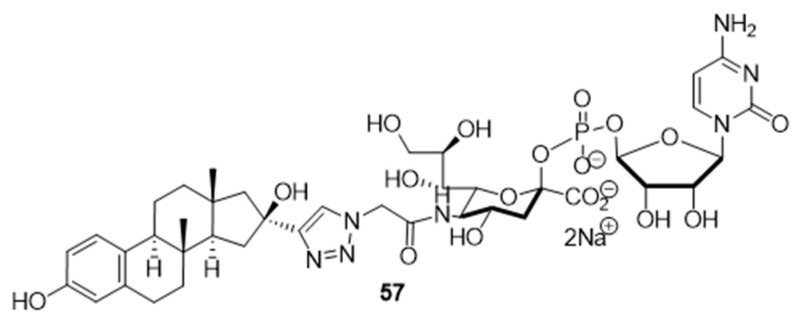
Structure of non-natural sugar triazole nucleotide **57** discovered from MS-based HTS.

At the current time, efforts are focused on the rapid identification of potent protein inhibitors (including ST inhibitors) using high-throughput screening (HTS) approaches. In this regard, the MS-based, rapid, and quantitative screening technique developed by Nishimura and coworkers is particularly attractive [145]. In this method, the inhibitory effects of substances on STs were determined quantitatively in the presence of CMP-Neu5Ac and an internal standard acceptor labelled with a stable isotope (OCD_3_). The assay was carried out by comparing the intensities of MS peaks of the expected products possessing OCH_3_ or OCD_3_ moieties in the absence and presence of inhibitors. A focused library of non-natural sugar triazole nucleotides was constructed by using click chemistry to link azidosugar nucleotides and various alkynes. Among the sugar triazole nucleotides prepared in this manner, **57**, possessing a steroid moiety, intriguingly displayed the highest inhibitory activity with an IC_50_ value of 8.2 μM against rST3Gal-III (Figure 10). It is very interesting that **57** was also accepted as a substrate (K_m_ = 125 μM) of rST6Gal-I [145]. It is most likely that multiple mechanisms are operating in the substrate and inhibition functions of the corresponding STs in living cells. Owing to this observation, the mechanism of **57** to suppress ST activity inside the cell remain unexplored. It is also worth mentioning that even very bulky substitutions (i.e., steroids) at the 5- or 9-position of the sialic acid portion did not hinder the sialylation reaction.

The difficulty of developing membrane permeable donor nucleotide analogues has been demonstrated to be a consequence of their highly hydrophilic and negatively charged nature. However, previous studies have shown that glycosyltransferase-mediated donor nucleotide biosynthetic pathways in cells tolerate a range of sugar analogues whether of natural or unnatural origin [146,147]. These results suggest the potential of a new strategy for the design of glycosyltransferase inhibitors that are active in cells which focuses on promiscuous monosaccharide salvage pathways and metabolic feedback loops to globally shutdown the function of STs.

**Figure 11:**
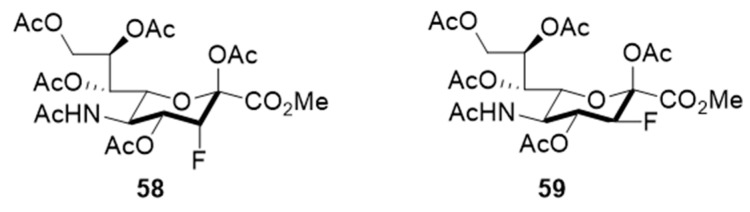
Structures of cell-permeable fluorine-substituted sialic acid analogues **58** and **59**.

In line with the observed relaxed substrate specificity of STs in sialic acid salvage pathways, the results of an investigation by Paulson and coworkers demonstrated that the global metabolic inhibitors of STs remodeled the glycome [137] because they are not used as a substrate but rather are bound to the active site of the enzyme. Specifically, it was found that peracetylated sialic acids **58**–**59**, bearing a fluorine at the position proximal to the endocyclic oxygen of the sugar moiety, were taken up and intracellularly metabolized to form the corresponding nucleotide sugar donor analogues (Figure 11). This activity effectively collapsed the synthesis of a range of sialylated glycan epitopes and altered the cellular glycome. The fact that membrane permeable fluorinated analogues of sialic acid are inhibitors of STs and have the potential to regulate glycosylation in vitro, might pave the way for the use of these metabolic inhibitors to explore the roles of sialylated glycans in cancers. As a matter of fact, compound **58** (3F_ax_-Neu5Ac) depleted α2,3- and α2,6-linked sialic acids in B16F10 cells, diminished migratory capacity, and reduced in vivo tumor growth [138]. As far as we know, this is the first in vivo investigation for sugar-based ST inhibitors and should pave way to further developments. The most recent report revealed that ST3Gal-VI inhibition using the pan-ST inhibitor **58** (3F_ax_-Neu5Ac) resulted in improved in vivo survival by increasing drug sensitivity. The ST inhibitor also suppressed the interaction of myeloma cells with E-selectin, MADCAM1, and VCAM1 and altered the post-translational modification of α4 integrin. In effect, sialyltransferase inhibition restricted myeloma cells from entering the protective bone marrow (BM) microenvironment wherein chemotherapeutic agents work less efficacious [148].

A recent development to the 3F_ax_-Neu5Ac inhibitor is the preparation of C-5-modified 3-fluoro sialic acid analogues [149]. Taking into consideration that the potency element of the compound depends on N-substitution, they replaced the natural N-acetamide group with a carbamate functionality which resulted in novel inhibitors **60**–**67** with much improved inhibitory activities and prolonged sialylation inhibition (Table 7). Very interestingly, all carbamate analogues **62**–**67** with facile modification were more potent than compound **60**. Compound **61** and **62** only differ in the α-substituent but the carbamate analogue exhibited a 56-fold increase in potency. This improvement was attributed to the more efficient metabolism of the designed inhibitors to their active CMP analogues, which in turn increased the effective inhibitor concentration inside the cells. It is worth mentioning that in spite of such potencies, there was no significant preference observed for the inhibition of α2,3-linked over α2,6-linked sialylation [149]. Nonetheless, further in vivo biological studies involving these new set of cell permeable ST inhibitors remain warranted. To this date, the actual downstream targets of these sugar-based inhibitors continue to be unresolved.

### 3.2. Non-Carbohydrate or Non-Sugar-Based ST Inhibitors

**Figure 12:**
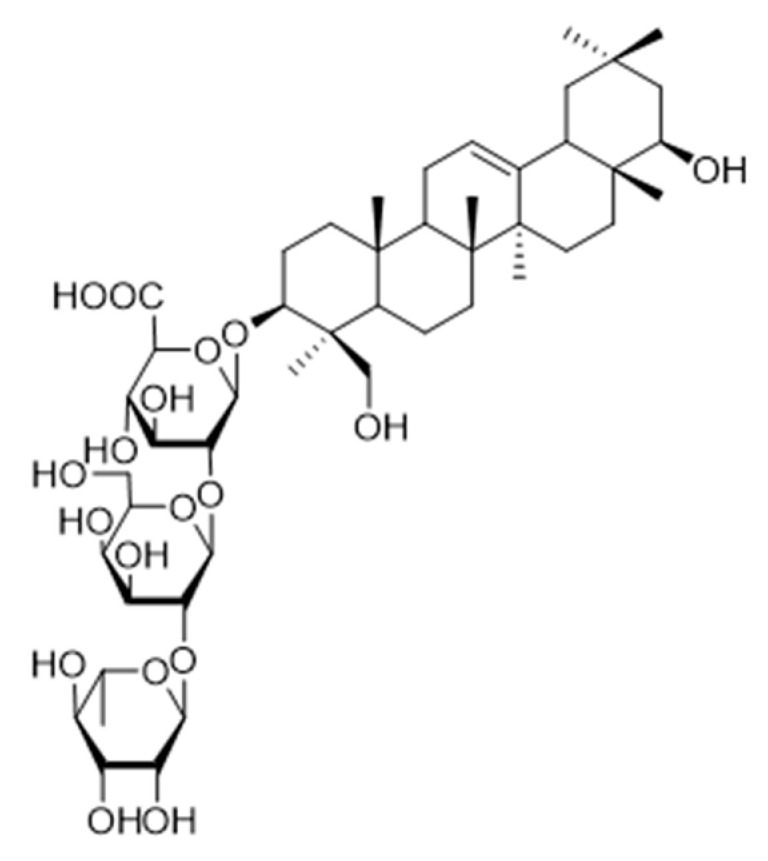
Structure of soyasaponin I.

One of the current challenges in developing effective ST inhibitors for clinical applications remains to be the poor membrane permeability of most small molecule candidates previously discussed. ST inhibitors obtained from natural sources are usually more lipophilic but are highly limited because of the lengthy screening processes and tedious chiral separations that are required to identify and purify desired targets. Despite these difficulties, Tsai and coworkers reported that soyasaponin I (Figure 12), a natural product derived from crude soybean saponin, which was identified by using a random screening process involving 7500 samples, acted as a highly specific inhibitor of ST3Gal-I (K_i_ = 2.3 μM) [150]. In addition, soyasaponin I selectively depressed mRNA expression of ST3Gal-IV and reduced tumor cell surface α2,3-sialic acid expression, resulting in the modification of the invasive behavior of tumor cells. In a highly metastatic cancer cell line B16F10, soyasaponin I effectively and specifically attenuated α2,3-sialylation on the cell surface, inhibited the migration ability of cancer cells, and enhanced cell adhesion to extracellular matrix proteins [151,152]. Treatment with soyasaponin I was found to cause a reduction of lung metastasis in mice, suggesting that the natural product altered sialylation of cell surface adhesion molecules bringing about a significant reduction in the ability of tumor cells to distribute to the lungs.

**Figure 13:**
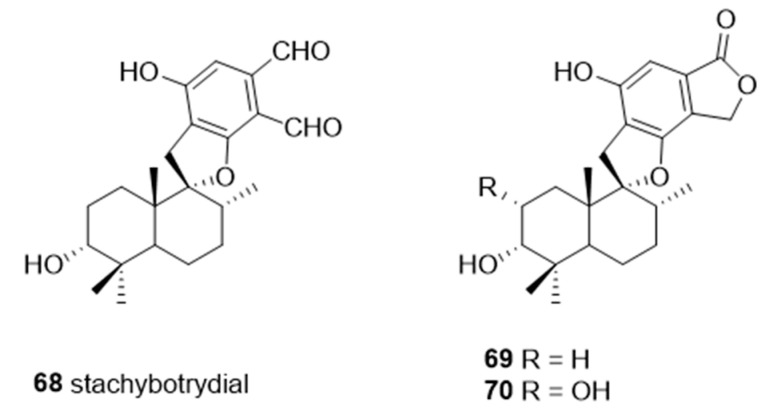
The structures of the spirocyclic drimanes, stachybotrydial **68–70**.

A naturally occurring member of the spirocyclic drimane family, stachybotrydial **68**, was isolated from the fungal strain, *Stachybotrys cylindrospora*, in 1993 [153]. Studies showed that **68** has inhibitory activities against cholesterol esterase [154], inositol monophosphatase [155], and avian myeloblastosis virus protease [156]. Among the spirocyclic drimanes tested, **68–70** (Figure 13) were shown to play diverse roles in inhibition of TNFα liberation from macrophage and antiplasmodial or antiviral activities [157,158]. Interestingly, the results of a study of sialyltransferase inhibition showed that **68**–**70** are potent inhibitors against various STs with IC_50_ values in the micromolar range (Table 8) [159]. These findings suggest that spirocyclic drimanes, and especially stachybotrydial, represent an interesting scaffold for the development of human ST inhibitors but may be impeded by their broad range of off-target effects and considerable synthetic inaccessibility.

**Figure 14:**
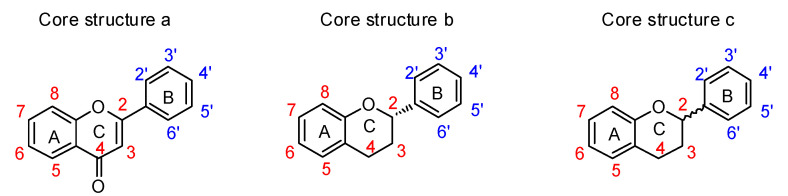
The core structures of flavonoid derivatives.

The utility of plant-derived flavonoids [160] as anti-tumor [161,162], anti-inflammatory [163], antibacterial [164], and antioxidant agents [165] has also been recognized. Members of this inhibitor family possess structures containing two phenyl groups (A-ring and B-ring) connected through three carbon atoms and one oxygen atom as part of a tetrahydropyran or dihydropyranone C-ring (Figure 14). Their broad range of biological activities suggest that flavonoids might also influence cancer-related processes. Suzuki and coworkers reported several flavonoid derivatives, containing three types of core structure (core a–c in Figure 14) and their results showed that some of these flavonoid analogues displayed significant inhibitory effects against rat ST6Gal-I, human ST6Gal-I, and rat ST3Gal-III (Table 9) [166]. Among the tested substances, **77** and **79** displayed the highest inhibitory activities, with IC_50_ values of 1.4–7.3 μM against the three STs.

Interestingly, the data revealed that most flavonoid analogues that exhibited ST inhibitory activity commonly have the benzopyranone core a, rather than core b or c framework, indicating that a double bond between C2–C3 in the C-ring (Figure 14) is essential for inhibitory activity. Furthermore, increasing the number of hydroxyl group on the B-ring of these substances, as exemplified by **77** versus **79** (Table 9), increased inhibitory activity. This observation implies that increasing the hydrophilic character of the B-ring leads to an enhancement of binding to sialyltransferases. In contrast, flavonoid analogues that contain a glucose group at C4′ of the B-ring are not ST inhibitors, suggesting that hydrophilic substituents larger than a hydroxyl group on the B-ring of the flavonoid backbone have an adverse effect on binding to STs. In addition, incorporation of a hydrophobic group such as methyl on A-ring hydroxyl groups of the flavonoids slightly enhanced inhibitory activity.

In view of the inhibitory activities displayed by flavonoids against sialyltransferases, the same group carried out studies to elucidate the nature of the inhibition against human ST6Gal-I by selected members of this series, including **71**, **76**, and **77**. A summary of the kinetic parameters arising from this effort is given in Table 10. The K_m_ value of recombinant human ST6Gal-I with CMP-Neu5Ac as the substrate was similar to that of the native enzyme reported elsewhere [167]. The K_m_ values of ST6Gal-I for CMP-Neu5Ac increased from 4.77 to 5.21 μM in the presence of 30 μM **71** and to 25.6 μM in the presence of 80 μM **71**. Studies using 75 μM **76** and 6 μM **77** showed the same trends in binding affinity (Table 10). In the presence of **71**, **76**, or **77**, the maximum rate of reaction (V_max_) values of ST6Gal-I decreased in a dose-dependent manner. These kinetic data suggest that these flavonoid analogues displayed a mixed type of inhibition of the sialyltransferases. Based on these findings, the flavonoid analogues discovered by Suzuki and coworkers are particularly attractive and may warrant further biological studies due to their lipophilic character, membrane permeability, and drug-like heteroaromatic scaffold.

**Figure 15:**
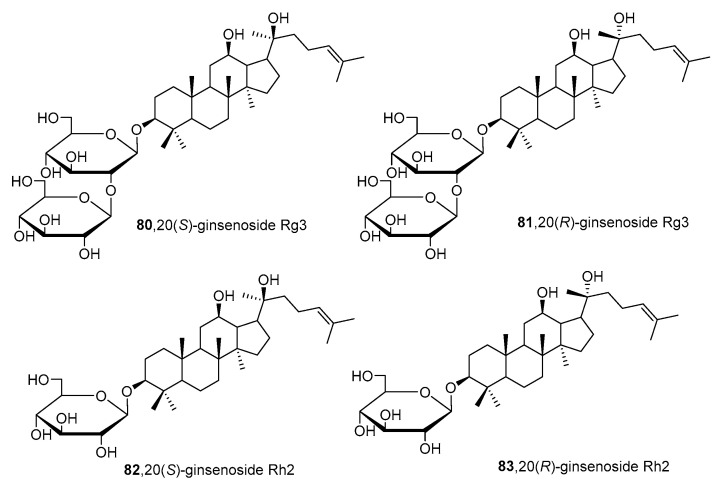
Structures of ginsenosides **80–83**.

Recently, ginsenosides **80**–**83** (Figure 15) isolated from the ethanolic extract of *Panax ginseng* C.A. Mey leaves were able to suppress total and free sialic acid expressions dose-dependently as well as inhibit ST expression [168]. From flow cytometry analysis, all compounds blocked sialylation of α2,3- and α2,6-linked sialic acids in HepG2 cells. In detail, compounds **82** and **83** with only one monosaccharide group possessed stronger inhibition attributed to their higher lipophilicity and permeability. Moreover, the *R* diastereomers were more inhibitory than their corresponding *S* counterparts, which was attributed to the difference in planarity which could have affected how the substrate attaches to the active site. Data further revealed that these ginsenosides showed partial selective inhibition of α2,6-sialylation compared to α2,3-sialylation [168]. However, further development of such natural product-based ST inhibitors remains limited by synthetic accessibility and availability.

Human bile acids (BAs), including primary BAs (cholic acid, chenodeoxycholic acid) and secondary BAs (deoxycholic acid, lithocholic acid), are oxidative metabolites of cholesterol in hepatocytes that form amphipathic derivatives with detergent-like, steroidal structures [169]. The results of several studies clearly showed that BAs not only modulated their own biosynthesis and their secretion through intracellular bile acid signaling pathways but that they also played an important role in other metabolic pathways. Bile acid receptors have been recognized to be targets for drug development, as exemplified by the observation that BAs have pro-apoptotic and pro-inflammatory activities [170]. Based on the fact that BAs showed in vivo potency in models for treatment of metabolic disorders, chronic liver diseases, hepatocellular cancer, and inflammatory diseases, we have examined the inhibition of STs involved in lung metastasis by lithocholic acid and other bile acids. This effort was inspired by the results of the studies with soyasaponin I, which possesses a pentacyclic ring system that is similar to the main skeleton of steroidal compounds. Epiandrosterone succinyl ester, a potent *Schistosoma japonicum* glutathione *S*-transferase inhibitor [171], and lithocholic acid, a substrate of nuclear pregnane X receptor [172], were identified through random screening of a library of steroidal compounds and shown to exhibit moderate inhibitory activities against ST3Gal-I with IC_50_ values of 350 and 21 μM, respectively. Our investigation has expanded the group of substances displaying ST inhibitory activity to include the epiandrosterone derivatives **84**–**86** and lithocholic acid derivatives **87–102**. Inhibition constants of these substances towards ST3Gal-I are listed in Table 11 [173]. Among these bile acid analogues, **102** was observed to have the highest inhibitory activity towards ST3Gal-I, with an IC_50_ of 5 μM. Analysis of a Lineweaver–Burk plot of the inhibition data showed that **102** is a noncompetitive inhibitor (K_i_ = 2.2 μM) towards the sialyl donor, cytidine monophosphate *N*-acetylneuraminic acid (CMP-Neu5Ac). However, the mode of inhibition of **102** towards the sialylation acceptor has not been determined thus far. Overall, these observations provide insight into the ST inhibitory pattern of lithocholic acid derivatives arising from soyasaponin I. Furthermore, these studies have pushed forward the development of ST inhibitors by providing a new structural family of substances that have cell-permeable properties and potential selective inhibition capability.

We have further conducted in-depth biological studies on the Lith-O-Asp steroid **93** because it is more synthetically accessible than other lithocholic acid derivatives (Table 11). Lith-O-Asp was found to exhibit inhibitory properties not only against ST3Gal-I, ST3Gal-III, and ST6Gal-I with IC_50_ values in the low micromolar range (12–37 μM), but also against the migration and invasion abilities of various lung cancer cell lines [174]. In addition, Lith-O-Asp also participated in anti-angiogenesis progression and decreased cell migration ability through inhibition of the integrin sialylation and downregulation of FAK/paxillin signaling pathway [174].

**Figure 16:**
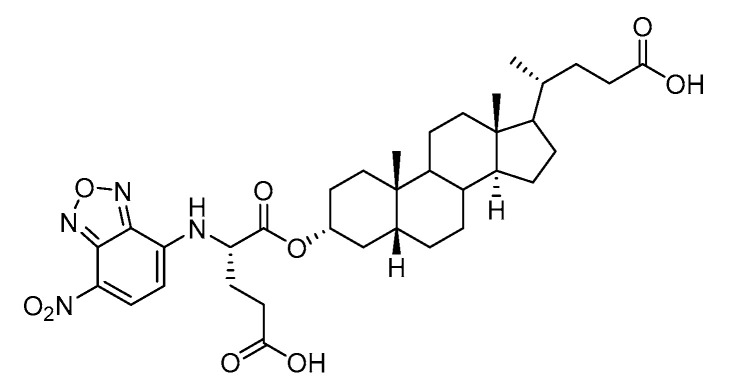
The structure of AL10.

AL10 (Figure 16), which possesses a lithocholic acid core structure containing a 7-nitro-2,1,3-benzoxadiazole (NBD) moiety, was initially discovered as a ST inhibitor in a structure-activity relationship (SAR) study. By effectively attenuating sialylation on the cell surface, cell-permeable AL10 inhibited adhesion, migration, actin polymerization, and invasion of α2,3-ST-overexpressing A549 and CL1-5 human lung cancer cells at nontoxic concentration levels [175]. Furthermore, studies with this substance suggested that AL10-induced inhibition of adhesion and migration is associated with reduced sialylation of various integrins that led to attenuation of activation of the integrin downstream signaling mediator FAK. Importantly, AL10 effectively repressed lung metastasis in experimental animals without negatively affecting liver and kidney function, highlighting the pertinence of natural bile acids as an antimetastatic scaffold.

In our exploitation of the lithocholic acid skeleton, we have regioselectively synthesized B- and C-ring-modified analogues with improved ST inhibitory activities [176]. Previous efforts have focused mainly on structural derivatization on the side chain carboxylic acid and the 3-α-OH groups, which led us to examine whether the steroid backbone influences the ST activity or not. In this particular work, we expanded the B and C rings via the insertion of -COO- or -CONH- to yield novel bile acid analogues **103**–**108** with lactone or lactam functionalities (Table 12). To our delight, compounds **103**, **105**, and **106** displayed greater potency with IC_50_ values equal to or below 3 μM. Moreover, moderate selectivity for N-sialylation was observed for these set of analogues as α2,3-(O)-sialylation was not inhibited in concentrations as high as 100 μM. Given the potency and selectivity observed for these compounds, further structural optimization and modifications are currently underway.

**Figure 17:**
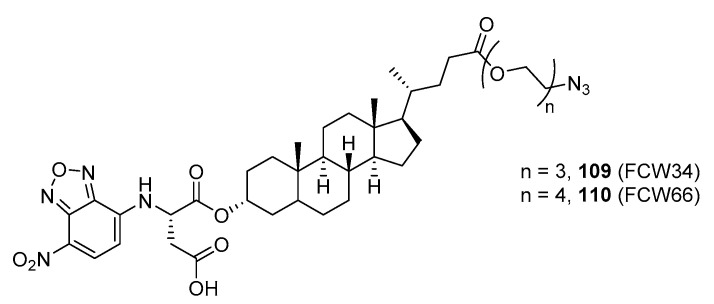
Chemical structures of two second-generation lithocholic acid-based inhibitors.

In the previous subsection, tight-binding substrate analogues have been developed but are generally hampered for use in biological and clinical applications due to their low lipophilicity. Another major challenge in the development of ST inhibitors that must be addressed is subtype-selectivity. Hence, the discovery of cell-permeable inhibitors that can target specific ST isozymes in vivo remains crucial for the development of novel antimetastatic drugs with less off-target and side effects. In our most recent work, we took on this challenge and were able to synthesize and biologically evaluate a second-generation series of cell-permeable and N- versus O-selective ST inhibitors [177]. Inspired by the previously reported pan-ST inhibitors (Lith-O-Asp and AL10) wherein we primarily focused on functionalization on the C3-α-OH group, we decided to divert our attention to derivatization at the cyclopentane ring side chain. To our delight, we discovered that conjugation of short-chain oligo(ethylene glycol) moieties to the carboxylic acid group led to a drastic improvement in subtype-selectivity (Figure 17). Among this series, the two most promising compounds **109** (FCW34) and **110** (FCW66) exhibited submicromolar IC_50_ values of 1.74 ± 0.09 μM and 1.01 ± 0.07 μM against α2,3-N-ST3Gal-III and 3.60 ± 0.40 μM and 4.90 ± 0.08 μM against α2,6-N-ST6Gal-I, respectively (Table 13). In contrast, compounds **109** and **110** displayed no inhibition at 500 μM and only 50–60% inhibition at 1mM against α2,3-O-ST3Gal-I which is indicative of the isozyme selectivity towards the two *N*-glycoprotein sialyltransferases. Further biological evaluation showed that the compounds inhibited breast cancer cell migration in a concentration-dependent manner, suppressed sialylation of integrin β subunits β1, β3, β4, and β5, hampered in vivo spontaneous metastasis, reduced in vivo tumor growth, and diminished angiogenic activity in vivo [175]. To the extent of our knowledge, this is the first report of biologically extensively studied ST inhibitors with cell membrane permeability, ST isozyme selectivity, and antimetastatic properties, thereby representing a major milestone in the ST-targeted cancer drug discovery and development.

**Figure 18:**
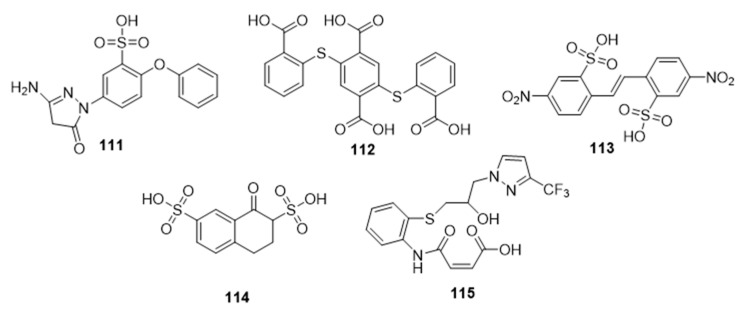
Structures of thioether and sulfonic acid derivatives **111–115**.

Facile and robust systems for high-throughput screening of sialyltransferase inhibitors that employ a fluorescence-polarization (FP)-based assay have also been developed by Paulson and coworkers [178]. The library tested using this method contained the five thioether and/or sulfonic acid derivatives, **111**–**115**, which were found to be inhibitors of STs (Figure 18, Table 14). One member of this group, **111**, exhibited potent inhibitory activity against ST3Gal-III with an IC_50_ value of 3.1 μM and a similar inhibitory potency towards ST3Gal-I and ST6Gal-I (IC_50_ = 14.1 and 10.8 μM, respectively) [178]. Intriguingly, **115** exhibited selective inhibitory activity against ST3Gal-III with an IC_50_ value of 1.7 μM, for it was a poor inhibitor of ST3Gal-I and ST6Gal-I (IC_50_ > 500 μM). Their findings offered alternative, non-sugar, potentially selective, and drug-like scaffolds for new classes of ST inhibitors. However, validation of their therapeutic effects in vitro and in vivo remains lacking.

## 4. Conclusions and Outlook

The past decades are a testament of the ever-growing biological evidence of the complex role of hypersialylation in cancer metastasis, inflammation, and related diseases. As discussed in this review, immense attention has emphasized the druggabiltiy of the sialyltransferase family as a target for the therapeutic treatment of cancer metastasis. Nonetheless, studies concerning ST3Gal-I and ST6Gal-I subtypes constitute the majority of what we currently know. Although we now know that STs may promote cancer metastasis by facilitating cell migration, invasion, and survival, as well as aiding immune evasion and chemotherapeutic resistance, it is still necessary to understand how the rest of the other human sialyltransferases affect and promote cancer metastasis. On the other hand, the pace of inhibitor design, synthesis, and development has remained generally slow. The development of the most potent nucleotide-based ST inhibitors has always been hampered by their poor membrane permeability and by the scarcity of in-depth in vivo biological and preclinical studies (Table 15). While it is true that a few pan-ST inhibitors (peracetylated 3F_ax_-Neu5Ac, soyasaponin I, lithocholic acid, and Lith-O-Asp) are now commercially available, the discovery of a subtype-selective ST inhibitor continue to be elusive. So far, the key discovery of FCW34 and FCW66 as N- versus O-selective ST inhibitors provide hope for site-specific N-type ST inhibition which may avoid unwanted disruptions on physiologically important O-sialylation processes and other off-target effects. However, the possibility of a dilemma between selectivity and efficacy should not be utterly disregarded. Sometimes, a very selective inhibitor will be less efficacious than a multitarget inhibitor because multitarget inhibitors are less prone to drug resistance since cancer metastasis is typically characterized by not just one but complex multi-dysfunctional signaling pathways. Thus, even with other FDA-approved chemotherapeutics for a specific type of cancer, a mixture of selective and multitarget inhibitors are usually observed.

At this time of writing, there are only a handful of reports utilizing rational (structure-based) drug design. This may be attributed to the unavailability of the protein structures of most human ST subtypes. To date, the only human ST isozymes with available crystal structures are ST8Sia-III, ST6Gal-I, ST6GalNAc-II. Future developments on the structural and molecular biology of STs will greatly benefit and considerably accelerate the design of inhibitors with improved isozyme selectivity and pharmacokinetic profiles. High-throughput screening (HTS) efforts also remain sparse, which may be a consequence of the sluggish innovation in ST inhibitor screening assays. Given our current pace in inhibitor design, HTS methods will all the more be pertinent in the future as the necessity for more synthetically accessible structural scaffolds increases.

To the best of our knowledge, there is no ST inhibitor currently undergoing clinical trials (clinicaltrials.gov). In this regard, we anticipate that a greater effort and attention will be devoted to this endeavor in the near future. Most of the ST inhibitors discussed in this review were interestingly non-cytotoxic, hence it is very intriguing to explore combination studies of ST inhibitors with other anticancer therapeutic strategies, given that several biological reports accentuated the potential use of ST inhibitors even for drug-resistant carcinoma cells. It is therefore not an overstatement to say that there is far more information that can be and has to be known to provide invaluable insights on and credence to sialyltranferase inhibition as a feasible therapeutic intervention to suppress cancer metastasis.

## Figures and Tables

**Table 1:** Selected upregulated sialyltransferase subtypes and their implications in cancer metastasis.

Sialyltransferase	Link to Cancer Progression and Metastasis	Reference(s)
**A.** **ST3Gal Family**		
ST3Gal-I	Upregulated in many cancer types (breast, ovarian, non-melanoma, and bladder cancer); overexpression promoted mammary tumorigenesis in mice and ovarian cancer cell migration, invasion (by inducing EMT), and conferred paclitaxel resistance	[32,33,34,35,44,51]
ST3Gal-II	Elevated ST3Gal-II mRNA levels observed in oral cancer with potential role in disease progression and metastasis	[50]
ST3Gal-III	Upregulated in different cancers (cervical, ovarian, oral, and breast); increased sLe^x^ expression, E-selectin adhesion, migration, and metastatic potential in pancreatic and breast adenocarcinoma cells; induced protein expression of metastasis-related proteins such as β1 integrin and matrix metalloproteinases (MMP-2 and MMP-9)	[36,37,38,39,45,46,47,50]
ST3Gal-IV	High ST3Gal-IV expression levels were observed in gastric and hepatic cancer cells; increased sLe^x^ expression, E-selectin adhesion, migration, and metastatic potential in ST3Gal-IV-overexpressing pancreatic cancer cells; induced sLe^x^ expression and c-Met activation in gastric carcinoma cells	[36,37,38,40,48,49]
ST3Gal-VI	Upregulation was associated with inferior survival in multiple myeloma; knockdown reduced cell adhesion and migration both in vitro and in vivo; increased expression levels promoted cell proliferation, migration, and invasion in liver cancer cells	[41,42]
**B.** **ST6Gal Family**		
ST6Gal-I	Upregulated in various carcinomas (colon, hepatic, non-melanoma, cervical, breast, ovarian, pancreatic, gastric, and non-small cell lung cancer) which correlated with distant metastasis, poor survival, EMT induction, and tumor invasion; known to induce β1 integrin-FAK mediated cell motility and migration as well as mediate invasiveness and tumorigenicity via the Notch1/Hes1/MMPs pathway; promoted chemoresistance and/or immune escape in several carcinoma cells	[13,43,44,45,49,52,53,54,63,64,65,66,67,68,69,70,71,72,73,74,75,76]
ST6Gal-II	High expression levels were associated with focal adhesion and metastasis pathways; downregulation inhibited levels of ICAM-1, VCAM-1, CD24, MMP2, MMP9, and CXCR4	[79]
**C.** **ST6GalNAc Family**		
ST6GalNAc-I	Overexpressed in breast and gastric cancer; responsible for the expression of sialyl-Tn (sTn) antigen which highly correlated with chemotherapeutic resistance and cancer metastasis	[81,83,84,85,86,87]
ST6GalNAc-II	Linked to the synthesis of sialyl-Tn (sTn) antigen associated with cancer aggressiveness; mediated follicular thyroid cancer cell invasion by regulating PI3K/Akt/NF-κB signaling pathway	[83,88]
ST6GalNac-V	Promoted breast cancer metastasis to the brain	[80]
**D.** **ST8Sia Family**		
ST8Sia-I	Upregulated in colon and breast cancer; mediated tumor growth and metastasis in triple-negative breast cancer; inhibition of ST8Sia-I led to suppression of FAK/Akt/mTOR and Wnt/β-catenin signaling pathways	[89,90,91,92,93]
ST8Sia-II	The highly invasive and migratory capabilities of glioma cells were found to be dependent on polySia expression	[96,97]

**Table 2:** Inhibitory activities of 5a’-carbadisaccharides **1**–**4** against α2,3- and α2,6-STs (Data from [106]).

#	Inhibitory Activities^a^ (IC_50_, μM)	
	α2,6-ST (rST6Gal-I)	α2,3-ST (rST3Gal-I)
**1**	903	419
**2**	533	185
**3**	651	245
**4**	>1 mM	>1 mM

^a^ 4-methylumbellipheryl LacNAc was used as the substrate with Michaelis-Menten constant (K_m_) values of 264 μM and 233 μM for α2,3- and α2,6-STs, respectively.

**Table 3:** Inhibitory potencies of 5′-triazole nucleosides **10–12** against α2,3-ST (Data from [123]).

Compound	#	IC_50_ (μM)	% Inhibition at 300 μM
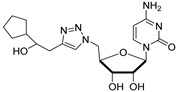	**10**	>300	44
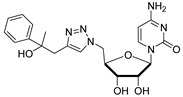	**11**	37.5	98
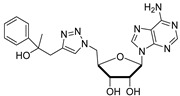	**12**	>300	8.6

**Table 4:** A new class of α2,3-ST Cst-06 inhibitors (Data from [125]).

#/% Inhibition at 500 μM										
First type										
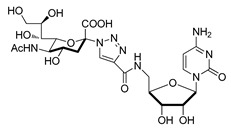						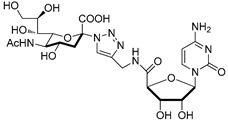				
**13**/51.7						**14**/58.5				
Second type										
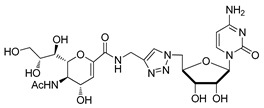						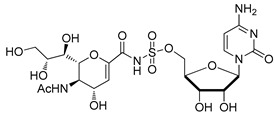				
**15**/29.2						**16**/33.5				
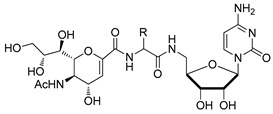										
R = H		R = (CH_2_)_4_NH_2_		R = (CH_2_)_2_CO_2_H			R = (CH_2_)_2_CONH_2_		R = 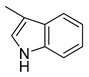	
**17**/10.5		**18**/10.7		**19**/30.5			**20**/19.1		**21**/25.1	
Third type										
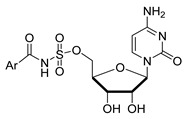										
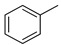	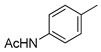		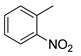		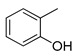			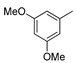		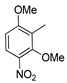
**22**/27.2	**23**/31.7		**24**/28.0		**25**/38.1			**26**/39.5		**27**/43.1
					
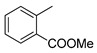	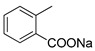		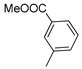		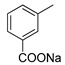			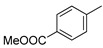		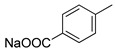
**28**/22.3	**29**/28.7		**30**/12.7		**31**/15.9			**32**/40.5		**33**/29.5
					
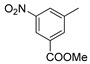	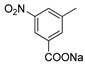		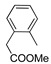		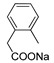			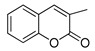		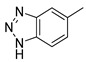
**34**/78.4	**35**/79.4		**36**/9.0		**37**/50.1			**38**/27.4		**39**/33.8

**Table 5:** Carbamate-linked uridyl-based hST6Gal-I inhibitors (Data from [134]).

Compound	#	% Inhibition at 10 μM	K_i_ (μM)
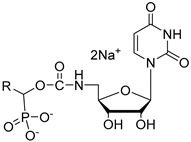			
R =			
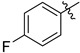	**46**	58.9	1.1 ± 0.1
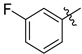	**47**	36.6	19.2 ± 2.1
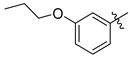	**48**	30.9	20.3 ± 2.0
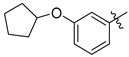	**49**	78.0	11.5 ± 5.6
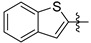	**50**	66.4	8.5 ± 1.0

**Table 6:** Inhibitory activities of **51**–**55** against α2,6- and α2,3-STs (Data from [136]).

#	Inhibitory Activities ^a^ (IC_50_, mM)	
	α2,6-ST (rST6Gal-I)	α2,3-ST (rST3Gal-I)
**51**	0.34	0.047
**52**	4.3	3.3
**53**	3.2	4.2
**54**	2.3	0.95
**55**	2.4	1.3

^a^ IC_50_ values at 0.11 mM CMP-Neu5Ac and 1.0 mM PA-LacNAc.

**Table 7:** Inhibition of α2,3-linked sialic acid in B16F10 cells by C-5-modified fluorinated sialic acid inhibitors **60–67** (Data from [149]).

Functional Group Family	R	#	EC_50_ (μM), B16F10 Cells
Amides	Methyl	**60**	26.8 ± 5.72
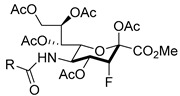	Butynyl	**61**	40.6 ± 5.67
Carbamates	Propargyl	**62**	0.72 ± 0.20
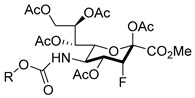	Allyl	**63**	1.39 ± 0.21
	Methyl	**64**	4.89 ± 0.25
	Ethyl	**65**	1.78 ± 0.16
	Isobutyl	**66**	3.33 ± 0.12
	Tert-butyl	**67**	3.48 ± 0.56

**Table 8:** IC_50_ values of spirocyclic drimanes **68–70** for different types of sialyltransferases (Data from [159]).

	Inhibition Activities IC_50_ (μg/mL)		
#	ST3Gal-I	ST3Gal-III	ST6Gal-I
**68**	6.7	10	0.61
**69**	30.2	50	8
**70**	168.4	110.5	18.5

**Table 9:** Summary of structures and inhibitory activities of flavonoids against three sialyltransferases (Data from [166]).

#	Functional Groups on the Flavonoid Core Structure											IC_50_ (μM)		
	C3	C4	C5	C6	C7	C8	C2′	C3′	C4′	C5′	C6′	rST6	hST6	rST3
**71**	H	=O	OH	H	OCH_3_	H	H	H	OH	H	H	39.0 ^A^	39.0 ^A^	39.0 ^A^
**72**	O-β-D-Glc	=O	OH	H	OH	H	H	H	OH	H	H	43.5 ^A^	-	-
**73**	H	=O	OH	H	O-β-L-Glc	H	H	H	OH	H	H	25.0 ^A^	40.0 ^A^	
**74**	H	=O	OH	β-D-Glc	OCH_3_	H	H	H	OH	H	H	32.9% ^B^	49.8% ^B^	10.7 ^B^
**75**	H	=O	OH	H	OC_2_H_5_	H	H	H	OH	H	H	93.5 ^A^	NT	NT
**76**	H	=O	H	H	H	H	H	H	OH	H	H	66.0 ^A^	81.7 ^A^	192.2 ^A^
**77**	H	=O	H	H	H	H	H	OH	OH	OH	H	5.1 ^A^	7.1 ^A^	1.4 ^A^
**78**	OH	=O	H	H	H	H	H	H	OH	H	H	424 ^A^	-	NT
**79**	OH	=O	H	H	H	H	H	OH	OH	OH	H	1.9 ^A^	7.3 ^A^	NT

rST6, hST6 and rST3 indicate ratST6Gal-I, human ST6Gal-I and rat ST3Gal-III, respectively. Glc, glucose. -, not inhibited significantly up to 50 μM compounds; NT, not tested. ^A^ IC_50_ values of compounds at 15 mM and 250 μM CMP-Neu5Ac for ST6Gal-I and ST3Gall-III, respectively. ^B^ Relative infectivity in the presence of compounds (50 μM) to control infection.

**Table 10:** Kinetic parameters of human ST6Gal-I in the presence of selected flavonoids (Adapted from [166]).

Compound 71			Compound 76			Compound 77		
Conc. (μM)	V_max_ (μM^−1^s^−1^)	K_m_ (μM)	Conc. (μM)	V_max_ (μM^−1^s^−1^)	K_m_ (μM)	Conc. (μM)	V_max_ (μM^−1^s^−1^)	K_m_ (μM)
0	0.66	4.77						
30	0.66	5.21	25	0.66	3.39	2.0	0.73	3.02
50	0.59	11.6	50	0.52	7.56	4.0	0.72	8.39
80	0.35	25.6	75	0.24	12.3	6.0	0.53	15.9

K_m_ values are for CMP-Neu5Ac in the presence or the absence of the flavonoids.

**Table 11:** Lithocholic acid-based α2,3-sialyltransferase inhibitors (Data from [173]).

#	Compound	IC_50_ (μM)	#	Compound	IC_50_ (μM)
**84**	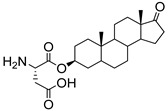	480	**94**	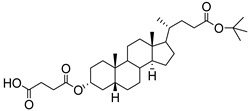	13
**85**	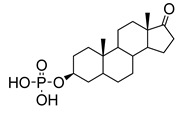	400	**95**	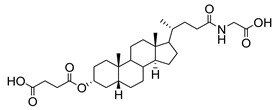	22
**86**	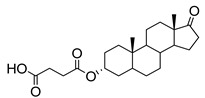	450	**96**	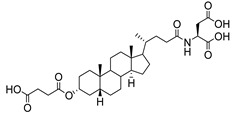	18
**87**	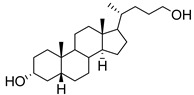	351	**97**	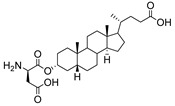	6
**88**	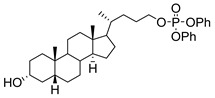	>100	**98**	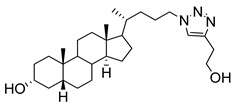	>100
**89**	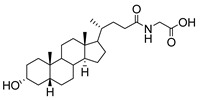	25	**99**	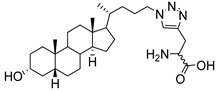	83
**90**	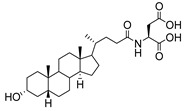	16	**100**	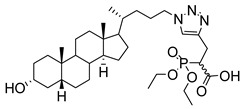	7
**91**	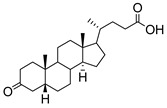	139	**101**	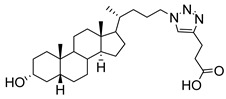	10
**92**	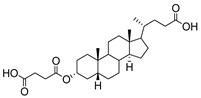	12	**102**	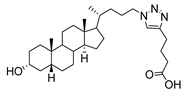	5
**93**	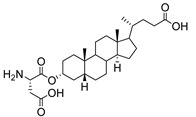	12			

**Table 12:** Inhibition of α2,6-(N)-ST and α2,3-(O)-ST by homolactones **103–105** and homolactams **106–108** (Data from [176]).

Compound	#	α2,6-(N)-ST (hST6Gal-I)		α2,3-(O)-ST (rST3Gal-I)	
		IC_50_, μM	% Inhibition at 3 μM	IC_50_, μM	% Inhibition at 100 μM
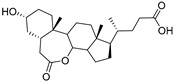	**103**	~3.0	50	>100	0
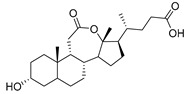	**104**	>3.0	44	>100	0
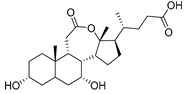	**105**	<3.0	54	>100	0
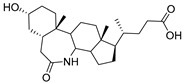	**106**	<3.0	57	>100	0
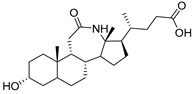	**107**	-	30	>100	0
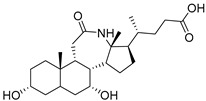	**108**	-	30	>100	0

**Table 13:** Inhibitory potency and selectivity of second-generation lithocholic acid-based ST inhibitors (Data from [177]).

Compound	#	IC_50_ (μM)			Selectivity Ratio	
		ST3Gal-III	ST6Gal-I	ST3Gal-I	α2,3-O/α2,3-N	α2,3-O/α2,6-N
FCW34	**109**	1.74 ± 0.09	3.60 ± 0.40	0% ^a^ (60%) ^b^	>287 ^c^	>139
FCW66	**110**	1.01 ± 0.07	4.90 ± 0.08	0% ^a^ (50%) ^b^	>495	>102
Lith-O-Asp	**93**	12.0	15.8 ± 0.10	39.0 ± 1.0	3	3
AL10	-	0.90 ± 0.1	1.50 ± 0.50	13.2 ± 0.6	15	9

^a^ Inhibition percentage was measured at 500 μM. ^b^ Inhibition percentage was measure at 1 mM. ^c^ The selectivity is expressed as the value of 500 μM/individual IC_50_ from ST3Gal-III without correction for the contribution of IC_50_ from ST3Gal-I.

**Table 14:** Inhibitory activities of HTS-discovered thioether and sulfonic acid derivatives **111–115** against three STs (Data from [178]).

#	Sialyltransferase IC_50_ (μM)		
	rST3Gal-III	pST3Gal-I	hST6Gal-I
**111**	3.1	14.1	10.8
**112**	8.2	10.7	133.5
**113**	4.1	133.2	56.0
**114**	4.0	63.5	106.4
**115**	1.7	>500	>500

**Table 15:** Selected promising sialyltransferase inhibitors as antimetastatic candidates.

Inhibitor Name (Compound #)	Chemical Structure	ST Inhibition	ST Subtype Selectivity	Preclinical Data	Clinical Data	Ref(s)
**I. Sugar-based**						
1. 48*l* (**40**)	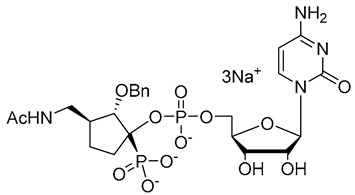	K_i_ = 0.028 μM (hST6Gal-I)	N.D. ^a^	N.D.	N.D.	[130]
2. **41**	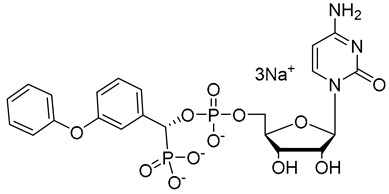	K_i_ = 0.019 μM (hSt6Gal-I)	N.D.	N.D.	N.D.	[119,130]
3. 5a-*s* (**42**)	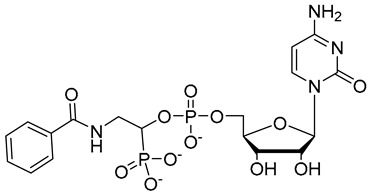	K_i_ = 0.016 μM (hSt6Gal-I)	N.D.	N.D.	N.D.	[131]
4. 28i-(*l*) (**49**)	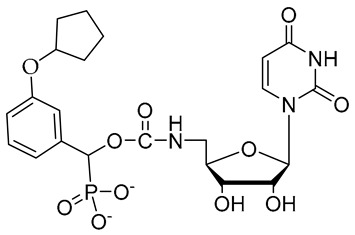	78.0% inhibition at 10 μM (hST6Gal-I); K_i_ = 11.5 μM	N.D.	N.D.	N.D.	[132]
5.peracetylated 3F_ax_-Neu5Ac (**58**)	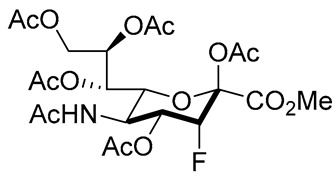	EC_50_ = 26.8 μM (α2,3-sialylation, B16F10 cells)	N.D.	Impaired cancer cell adhesion to ECM components, inhibited cell migration, and reduced tumor growth in vivo	N.D.	[137,138,148,149]
6. SiaFPoc (**62**)	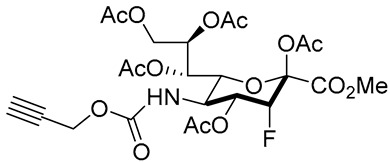	EC_50_ = 0.72 μM (α2,3-sialylation, B16F10 cells)	N.D.	N.D.	N.D.	[149]
**II. Non-sugar- based**						
7. Soyasaponin I	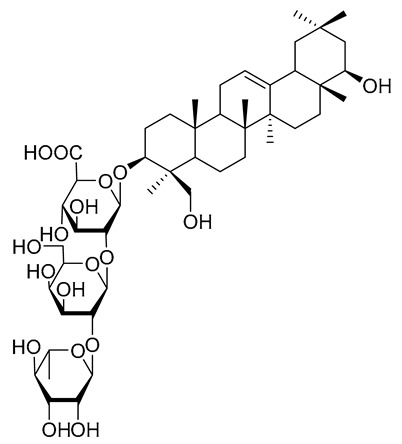	K_i_ = 2.3 μM (ST3Gal-I); inhibited the expression of α2,3-linked sialic acids on B16F10 cell surface	N.D.	Enhanced cell adhesion to ECM proteins and decreased the migration ability of B16F10 cells but showed no effect on cell invasiveness; reduced pulmonary metastasis in vivo	N.D.	[150,151,152]
8. Lithocholic acid	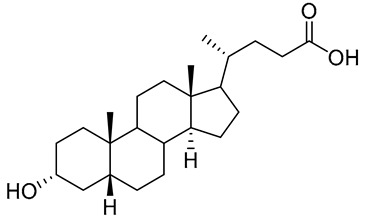	IC_50_ = 21 μM (rST3Gal-I)	N.D.	N.D.	N.D.	[173]
9. Lith-O-Asp (**93**)	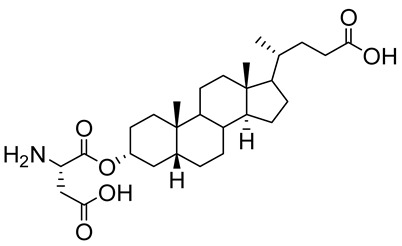	IC_50_ = 37 μM (rST3Gal-I), 15.8 μM (hST6Gal-I), 12.2 μM (hST3Gal-III)	Non-selective	Inhibited migration and invasion abilities of lung cancer cells (A549, H1299, CL1-5) and breast cancer cells (4T1-Luc) in vitro; reduced lung metastasis in vivo; suppressed tube formation in HUVEC and upregulated angiogenic inhibitors (RNH1 and PGK1); inhibited the integrin/FAK/paxillin signaling pathway and its corresponding downstream effectors	N.D.	[173,174]
10. AL10	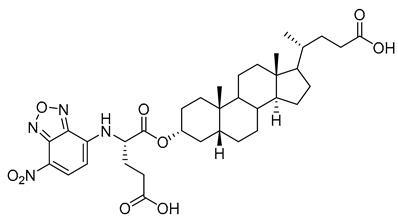	IC_50_ = 0.88 μM (rST3Gal-I), 1.50 μM (hST6Gal-I)	Non-selective	Inhibited adhesion, migration, actin polymerization and invasion of α2,3-ST-overexpressing A549 and CL1-5 cells; suppressed lung metastasis in vivo	N.D.	[175]
11. FCW34 (**109**)	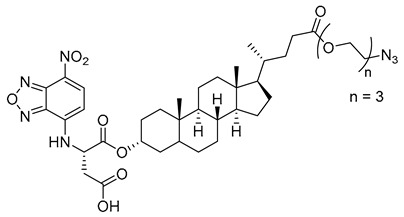	IC_50_ = 1.74 μM (hST3Gal-III), 3.60 μM (hST6Gal-I), >500 μM (rST3Gal-I)	ST3Gal-III and ST6Gal-I selective	Diminished migration ability of MDA-MB-231 cells (IC_50_ = 10.64 μM); attenuated sialylation of integrin β1, β3, β4, and β5; exhibited in vivo metastasis inhibition and decreased tumor growth; inhibited talin/integrin/FAK/paxillin and integrin/NFκB signaling pathways; lessened neovascularization in a transgenic zebrafish model	N.D.	[177]
12. **115**	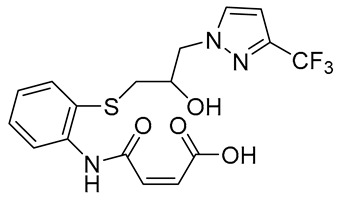	IC_50_ = 1.7 μM (rST3Gal-III), >500 μM (pST3Gal-I and hST6Gal-I)	ST3Gal-III- selective	N.D.	N.D.	[178]

^a^ N.D. = Not determined.

## Data Availability

No new data were created or analyzed in this study. Data sharing is not applicable to this article.
